# Synaptojanin-1 supports VPS35-dependent trafficking of dopamine D2 autoreceptors at presynaptic terminals

**DOI:** 10.21203/rs.3.rs-7730224/v1

**Published:** 2025-10-15

**Authors:** Nirmal Kumar, Elnaz Khezerlou, Justin Cai, Hanna Caiola, Ulrik Gether, Huaye Zhang, Ping-Yue Pan

**Affiliations:** 1 Department of Neuroscience and Cell Biology, Rutgers University Robert Wood Johnson Medical School, 675 Hoes Lane West, Piscataway, NJ 08854, USA; 2 Graduate Program in Neuroscience, Rutgers University; 3 Department of Neuroscience, University of Copenhagen, Blegdamsvej 3, DK-2200 Copenhagen, Denmark

**Keywords:** synaptojanin1, presynaptic sorting, dopamine D2 autoreceptor, VPS35, Parkinson’s disease

## Abstract

Synaptic dysfunction is a hallmark of early Parkinson’s disease (PD), but molecular mechanisms underlying dopaminergic synaptic impairment and vulnerability remain poorly understood. Here, we identify a functional interaction between two PD genes, Synaptojanin1 and VPS35, in regulating endosomal sorting of the dopamine D2 short (D2S) autoreceptor, thereby modulating dopamine signaling. We show that Synaptojanin1 deficiency results in intracellular retention of the D2S, impaired gating of dopamine release and reduced behavioral responsiveness to a D2-like agonist. VPS35 is recruited to D2S-containing and Rab7a-positive endosomes in a Synj1-dependent manner, and VPS35 overexpression overcomes Synj1 deficiency-associated impairment in D2S surface delivery in axons. Moreover, Synj1 regulates VPS35 expression and localization in dopaminergic axons, indicating their broader roles in presynaptic cargo sorting. These findings reveal a novel cooperation between a synaptic vesicle endocytic regulator and a core component of the retromer complex, providing new mechanistic insights into presynaptic trafficking and its disruption in PD.

## Introduction

Parkinson’s disease (PD) is a prevalent neurodegenerative disorder characterized by the progressive loss of dopaminergic neurons in the substantia nigra. Emerging evidence suggests that dopaminergic synaptic dysfunction is a hallmark of early PD that occurs prior to neurodegeneration^1–6^. Many PD-associated genes, such as Synaptojanin1 (Synj1), Auxilin1, and Endophilin A, are known to regulate presynaptic vesicle recycling^7–23^, however, the mechanisms underlying the maintenance of the dopamine synapse remain elusive.

Presynaptic terminals are sites of active membrane turnover due to the presence of synaptic vesicles, which undergo spontaneous and activity-dependent recycling. This busy traffic requires molecular machineries to operate at different time scales to maintain membrane protein (cargo) composition and to ensure protein quality against use-dependent decline^24–27^. Recent studies point out the novel roles of synaptic vesicle endocytic proteins in quality control at the presynaptic terminal. For example, autophagosome formation has been shown impaired in the *Drosophila* and *C.* elegans synapses expressing Synj1^28,29^ or Endophilin A^30,31^ mutants. Deletion of Auxilin1 results in sorting defects affecting the synaptic vesicle proteome and locomotor deficits in mice^32^. Studies from us and others further show that Synj1 regulates the abundance of surface receptors and transporters^33–35^, suggesting its potential role in endosomal sorting and delivery of surface cargos to maintain the plasma membrane.

At neuronal soma, endosomal sorting and delivery of surface cargos are thought to depend primarily on the retromer complex^36–40^, which contains VPS35 – another PD associated gene^41–44^. Deletion of VPS35 has been shown to affect the surface expression of postsynaptic receptors^37,39,40,42^. However, sorting at the presynaptic terminal and its impact on neurotransmitter release is less understood. Recent studies showed that presynaptic μ-opioid receptors^45^ and dopamine transporters^46–48^ are localized to VPS35-positive endosomes, suggesting VPS35 could regulate presynaptic function and neurotransmitter dynamics. However, conflicting results have been reported at different types of synapses. Notably, deleting or mutating VPS35 did not affect glutamate release^38,49^, but affected dopamine release^50,51^, suggesting an important role of VPS35-dependent endosomal sorting for the function of the dopamine synapse.

Dopamine release is regulated by synaptic vesicle fusion, recycling, and gated by the dopamine D2 receptor short isoform (D2S). The D2S is a Gα_i/o_-coupled class-A GPCR that functions as an autoreceptor to inhibit dopamine release^52,53^. Despite the relevance of D2 receptors in various neurological disorders, including substance abuse^54–56^, schizophrenia^57,58^, as well as PD^59–61^, the regulation of its turnover at the cell surface remains controversial^62–66^. The molecular machinery involved in regulating presynaptic D2S is poorly understood.

In this study, we uncover a novel functional interaction between the core retromer component, VPS35 and the synaptic vesicle endocytic molecule, Synj1, in regulating presynaptic D2S receptor surface expression and dopamine synapse function. We provide evidence that Synj1 supports the endosomal sorting of D2S through the VPS35 retromer pathway, influencing dopamine signaling both *in vitro* and *in vivo*. Synj1 deficiency leads to impaired VPS35 expression and recruitment to endosomal D2S, while VPS35 overexpression rescues the impaired D2S surface delivery in Synj1 deficient dopaminergic axons (Supplemental Fig. 1). We further found correlated expression and co-localization of Synj1 and VPS35 in dopaminergic axons, suggesting a cooperative role in supporting broader types of surface cargos beyond D2S. These findings reveal novel molecular underpinnings of presynaptic sorting, which might be crucial in maintaining dopaminergic synapses in early PD pathogenesis.

## Results

### *Synj1*+/− male mice display a reduced behavioral sensitivity to a D2-like agonist.

**Fig. 1:**
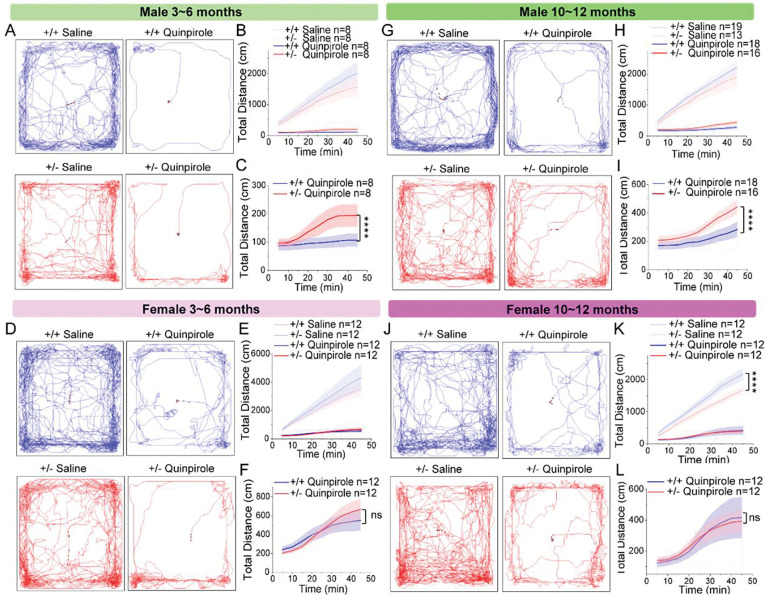
*Synj1*+/− male mice exhibit reduced behavioral sensitivity to a D2-like agonist. **A-F**) *Synj1*+/+ and *Synj1*+/− littermate male **(A-C)** and female **(D-F)** mice at 3–6 months of age were subject to i.p. injection of saline or a D2-like agonist, quinpirole 0.5 mg/kg. Mice activity in total travel distance was monitored for 45 min in an open field chamber. Cumulative distance was plotted against time, and quinpirole groups were presented in a different scale in **C** for the male cohort and **F** for the female cohort. **G-L**) A similar study was carried out for a separate older cohort of littermate male (**G-I**) and female (**J-L**) mice at 10–12 months of age. Data = mean ± S.E.M. n = animal number. ns = non-significant, ****p<0.0001, two-way ANOVA-RM.

To gain insight into whether Synj1 regulates dopamine D2 receptor-mediated signaling, we prepared a young (3–6 months) cohort and an older (10–12 months) cohort of male and female *Synj1*+/+ and *Synj1*+/− littermate mice. Mice were allocated into two groups that received intraperitoneal injection of saline or the D2-like agonist, quinpirole. Given the reported biphasic effects of quinpirole^67,68^, we used a moderate dose (0.5 mg/kg^69,70^) and recorded the total travel distance over 45 min. All quinpirole-treated mice exhibited substantially lower locomotor activity compared to the saline mice. The locomotor-suppressing effect of quinpirole was attenuated in male *Synj1*+/− mice relative to male wild-type littermates at both ages (Fig. 1A–C, G–I). In contrast, quinpirole suppressed locomotor activity to a similar degree in female *Synj1*+/− mice and wild-type mice (Fig. 1D–F, J–L). Together, these findings suggest that Synj1 is involved in D2 receptor control of locomotor activity in male mice. The attenuated response to D2 receptor stimulation in *Synj1*+/− mice perhaps reflects an early pathological change prior to the loss of striatal dopamine and broader locomotor deficits at 12 months as we showed previously^71^.

### Dopamine release sites in *Synj1*+/− neurons exhibit reduced D2 autoreceptor-mediated release inhibition.

**Fig. 2:**
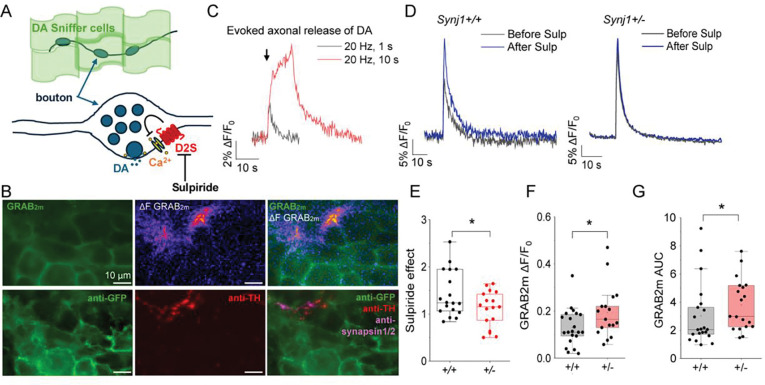
Dopamine release sites in *Synj1*+/− neurons exhibit reduced disinhibition to a D2-like antagonist. **A**) Illustration of the experimental setup to measure axonal dopamine release in the ventral midbrain neuron-dopamine sniffer cell coculture. The Ca^2+^-dependent dopamine release from the bouton (release site) is regulated by basal D2S mediated inhibition. Local DA release can be detected by GRAB_DA2m_ dopamine sniffer cells seeded on top. **B**) Representative images of electrically stimulated dopamine release from axonal release sites from the coculture. Top, live image of GRAB_DA2m_ at baseline, the ΔF image of the GRAB_DA2m_ response to a 20 Hz, 1 s field electrical stimulation, and the overlay of the two images. Bottom, *post-hoc* immunofluorescence of the sniffer cells labeled by anti-GFP, the underlying dopaminergic axons labeled by anti-TH, and the overlay of the two channels with an additional marker for synapsin1/2 (indicate dopamine release site). **C**) Representative traces of GRAB_DA2m_ response to a 20 Hz, 1 s (black) and a 20 Hz, 10 s (red) stimulations for the same release sites. **D**) Representative traces of the GRAB_DA2m_ response from *Synj1*+/+ and *Synj1*+/− littermate cultures to a 20 Hz, 1 s stimulation before (black) and after (blue) a 2-min incubation of a D2-like antagonist, sulpiride 5 μM. **E-G**) Summary of the sulpiride effect on peak release potentiation (**E**) the GRAB_DA2m_ ΔF/F_0_ peak (**F**), and area under the curve (AUC) (**G**) in *Synj1*+/+ (n = 18 release sites) and *Synj1*+/− (n = 17 release sites) littermate neuronal cultures. Data = mean ± S.E.M. from 5 batches of cocultures. *p<0.05, Student’s *t*-test.

There are two isoforms of D2 in the brain – D2L and D2S – that could contribute to the blunted locomotor-suppressing effect of quinpirole in male *Synj1*+/− mice. Previous studies showed that quinpirole-induced locomotor inhibition was unchanged in D2L deletion mice compared to wildtype mice^70,72^. This suggests that D2L may not explain the loss of quinpirole sensitivity in *Synj1*+/− mice. To investigate the involvement of D2S, we first conducted an imaging analysis to assess the contribution of D2S to the evoked bouton release of dopamine in cultured *Synj1*+/− neurons. We used a ventral midbrain-dopamine sniffer cell coculture system to detect evoked dopamine transients at neuronal boutons^35^. Sniffer cells with stable expression of GRAB_DA2m_^73–75^ were seeded onto days *in vitro* (DIV) 13 ventral midbrain neuron cultures from littermate *Synj1*+/− and *Synj1*+/− pups (Fig. 2A–B). Sniffer cells reach ~80% confluency two days after seeding when dopamine measurements were conducted. Field electrical stimulations reliably induced fluorescence peaks triggered by dopamine release from presynaptic boutons (Fig. 2B–C). To assess D2 autorecepotor (D2S) function, we applied a D2-like antagonist, sulpiride (5 μM), and measured the evoked dopamine release before and after for comparison of the disinhibitory effect (Fig. 2A, D). Sulpiride increased the amplitude of the evoked GRAB_DA2M_ response in *Synj1*+/+ neurons by 40% without affecting the baseline (Supplemental Fig. 2A). A significantly smaller effect (10%) was found in *Synj1*+/− neurons (Fig. 2D, E), indicating impaired D2S function in Synj1 deficient neurons. The evoked GRAB_DA2M_ response before sulpiride was also larger in *Synj1*+/− neurons compared to control (Fig. 2F–G), consistent with deficits in endogenous D2S expression and/or function. The significantly higher baseline GRAB_DA2m_ fluorescence and a trend of faster decay in the evoked responses in *Synj1*+/− neurons (Supplemental Fig. 2) were largely in line with our previous findings reflecting an altered DAT function^35^.

### Basal axonal D2S surface fraction is not altered in *Synj1*+/− neuronal axons.

**Fig. 3:**
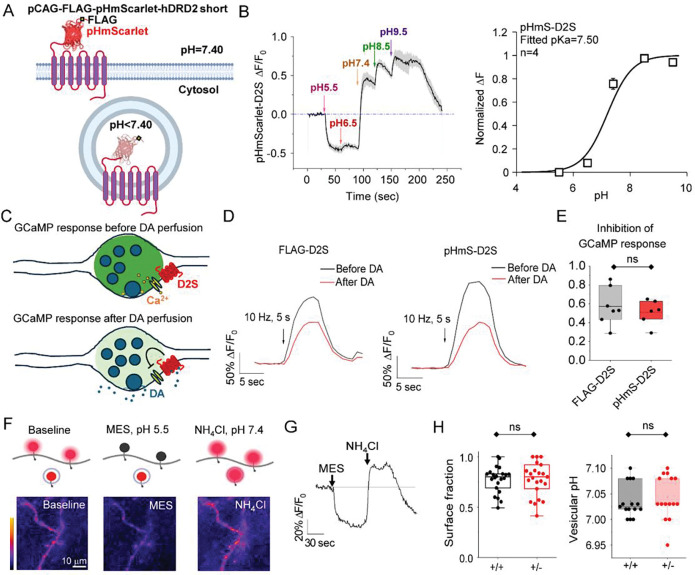
Basal axonal D2S surface fraction is not altered in *Synj1*+/− neuronal axons. **A**) Illustration of the molecular engineering and working mechanism of pHmScarlet-D2S. The pHmScarlet is a red-shifted pH sensitive fluorescent protein. It fluoresces at the neutral pH on the cell surface and becomes dimmer when inside an acidic vesicle. **B**) Measurement of the pKa of pHmScarlet-D2S at neuronal axons. Axons expressing pHmScarlet-D2S were sequentially perfused with Tyrode’s solutions buffered at various pH. Data = mean ± S.E.M. n = 4 independent experiments. The dynamic fluorescence change was rescaled to 1 and the relative ΔF at each pH was fitted by the Henderson-Hasselbach equation to find the fitting pka = 7.50. **C**) Cartoon illustrating the experimental design by measuring the dopamine (DA)-induced inhibition of presynaptic Ca^2+^ to determine the function of the tagged D2S. (**D-E**) Representative GCaMP traces for the same set of boutons co-expressing either FLAG-D2S or pHmScarlet-D2S (**D**) and the comparison for their DA-induced inhibition (**E**). p >0.05 from Student’s *t-*test **F)** Cartoon illustrating the fluorescence change (top) and representative images of an axon expressing pHmScarlet-D2S (bottom) during perfusion of a membrane impermeable acid buffer, MES (pH5.5) and a membrane permeable neutralizing solution NH_4_Cl (pH7.4). **G**) Representative pHmScarlet-D2S fluorescence trace at axons following a sequential perfusion of MES and NH_4_Cl as in **F**. **H**) Summary of the basal surface fraction and D2S-containing vesicular pH measured by the MES and NH_4_Cl perfusion method and calculated using the Henderson-Hasselbach equation. Data from 4 batches of independent littermate cultures. *Synj1*+/+ (n = 22) and *Synj1*+/− (n = 23). p >0.05 from Student’s *t-*test.

The reduced sulpiride effect in *Synj1*+/− neurons could be due to receptor loss of function or lack of receptor on the surface. Currently, there is no specific and reliable labeling method for endogenous axonal D2S. To determine the surface D2S level, we constructed a novel pH-sensitive reporter by fusing a red-shifted variant of pHluorin, pHmScarlet^76^, to the extracellular N-terminus of human FLAG-D2S (Fig. 3A). This pHmS-D2S biosensor (pCAGpromotor-FLAG-pHmScarlet-hD2S) fluoresces at neutral pH on the cell surface and quenches in acidic intracellular compartments, enabling measurements of the dynamic localization of the axonal D2S (Fig. 3A). By perfusing a series of differentially buffered Tyrodes’ solution at various pH (Fig. 3B), we determined the pKa of the newly constructed pHmS-D2S to be 7.50, similar to the reported ideal pKa of 7.40^76^. To determine whether the pHmS-tagged D2S receptor remains functionally intact, we evaluated its effect on presynaptic voltage-gated Ca^2+^ channels (VGCC) by comparing it to the FLAG-D2S (Fig. 3C–E). Activation of D2S by dopamine perfusion leads to Gα_i/o_-coupled inhibition of VGCC, and the reduction in presynaptic Ca^2+^ will be taken as a readout of D2S function (Fig. 3). The Ca^2+^ sensor, GCaMP6f were co-expressed with pHmS-D2S or FLAG-D2S in cultured ventral midbrain neurons. GCaMP responses to electrical stimulation were measured before and after perfusion with 10 μM dopamine (Fig. 3D). We found similar dopamine-induced reductions in GCaMP responses between neurons expressing pHmS-D2S and those expressing FLAG-D2S (Fig. 3D, E). Together, these findings confirm that pHmS-D2S exhibits pH sensitivity and endogenous functions in dopamine sensing, allowing us to use this tagged receptor as a tool to assess the dopamine-regulated trafficking of D2S in live neurons.

We expressed pHmS-D2S in primary midbrain neurons derived from *Synj1*+/+ and *Synj1*+/− mice to quantify their D2S surface fraction. As shown before^35,77,78^, neurons were sequentially perfused with a membrane impermeable MES buffer (pH = 5.50) to quench surface fluorescence, followed by an NH_4_Cl buffer (pH = 7.40) to neutralize acidic compartments and reveal total D2S signal (Fig. 3F, G). The surface fraction was calculated based on the normalized fluorescence peaks of MES and NH_4_Cl perfusions using the Henderson-Hasselbach equation. Surprisingly, *Synj1*+/− neuronal axons showed no significant difference in D2S surface fraction or intracellular D2S vesicle pH at baseline compared to *Synj1*+/+ axons (Fig. 3H). Although this finding could be due to receptor overexpression masking differences of the endogenous D2S, it could also suggest that the reduced D2 pharmacological effects in *Synj1*+/− mice and neurons are due, in part, to the desensitization of surface D2S.

### Basal axonal D2S surface fraction is reduced in *Synj1*-depleted dopamine neurons.

**Fig. 4:**
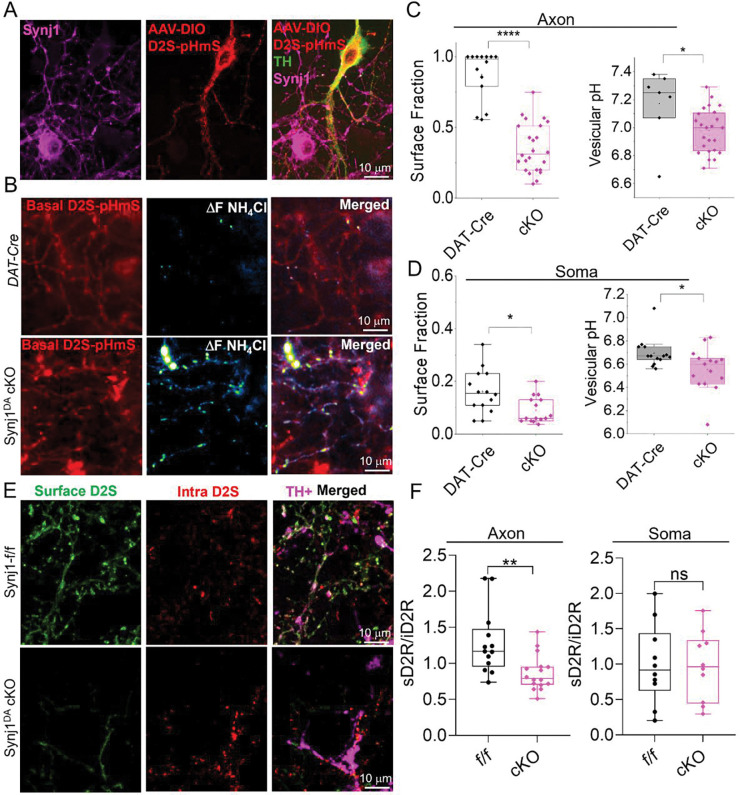
Basal axonal D2S surface fraction is reduced in Synj1 depleted dopamine neurons. **A)** Immunofluorescence images of AAV9-SYNp-DIO-pHmScarlet-FLAG-D2S specifically expressed in cultured dopaminergic neurons from the *Synj1*^DA^ cKO mice. **B)** Basal pHmScarlet-D2S fluorescence and the ΔF of the NH_4_Cl perfusion response in *DAT-Cre* and *Synj1*^DA^ cKO neuronal axons expressing AAV-SYNp-DIO-pHmScarlet-D2S. The NH_4_Cl ΔF response indicates the abundance of intracellular D2S. **C-D)** Summary of the axonal (**C**) and soma (**D**) pHmS-D2S surface fraction (****p <0.0001 for axon, *p= 0.017 for soma, Mann-Whitney tests) and vesicular pH (*p = 0.019 for axon, *p= 0.023 for soma, Mann-Whitney tests) in *DAT-Cre* (n =17 axons, n=13 soma) and *Synj1*^DA^ cKO (n=24 axons, n=14 soma) DA neurons. **E-F**) Immunofluorescence analysis for surface (without membrane permeabilization) and intracellular (after membrane permeabilization) D2S in *Synj1*^*f/f*^ and *Synj1*^DA^ cKO dopamine neurons expressing FLAG-D2S. *Synj1*^*f/f*^ (n= 13 for axon, n=10 for soma) and *Synj1*^DA^ cKO (n = 16 for axon, n = 10 for soma) from 2 batches of cultures. **p = 0.0036 for axon and p = 0.90 for soma, Student’s *t*-test.

The observation in *Synj1*+/− neurons prompted us to examine whether complete loss of *Synj1* affects D2S surface availability in dopamine neurons. Mice with *Synj1* complete deletion is perinatally lethal. To more effectively analyze D2S surface expression in *Synj1* deleted dopamine neurons, we generated a condition-ready mouse line (*Synj1*^*flox/flox*^) with *loxP* sites inserted to both ends of the critical exon 4. When crossed to the *DAT*^*IRESCre*^ (*DAT-Cre*) driver line, offspring (*Synj1*^*DA*^ cKO mice) exhibit complete deletion of *Synj1* in all dopamine neurons, which was verified in our ventral midbrain cultures (Fig. 4A). To determine the surface fraction of D2S in these neurons, we transduced the *Synj1*^*DA*^ cKO culture or control *DAT-Cre* culture with AAV9-hSynapsin promotor-DIO-pHmS-D2S and performed the MES and NH_4_Cl perfusion experiments. We observed a significant reduction in D2S surface fraction and an increased presence of intracellular D2S in *Synj1*^*DA*^ cKO neurons (Fig. 4B–C). Consistently, the intracellular D2S vesicles exhibited lower pH, suggesting their altered trafficking. To independently validate these findings, we performed immunofluorescence analysis for neurons expressing FLAG-D2S. We found that the extracellular-to-intracellular anti-FLAG fluorescence was significantly higher in *Synj1*^*flox/flox*^ neuronal axons compared to those in *Synj1*^*DA*^ cKO neurons, although a difference at the soma was not detected (Fig. 4E–F). Together, these results demonstrate that Synj1 is required to maintain basal D2S receptor surface level in dopaminergic axons.

### Repeated dopamine exposure leads to maladaptive D2S receptor trafficking and a greater reduction at bouton surface in *Synj1*+/− neurons.

**Fig. 5:**
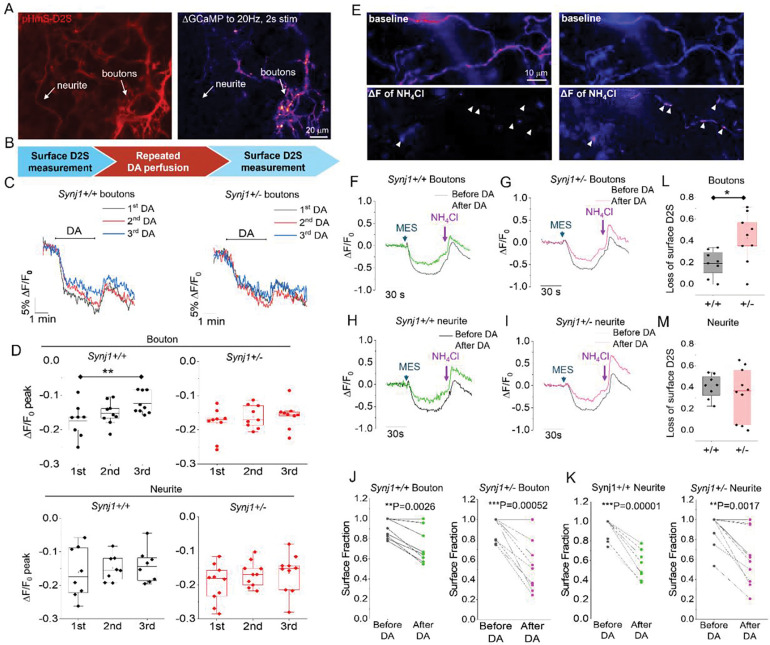
Repeated dopamine exposure leads to maladaptive D2S receptor trafficking and a greater reduction at bouton surface in *Synj1*+/− neurons. **A)** Representative images of ventral midbrain neuronal processes expressing pHmScarlet-D2S and GCaMP6f. The ΔF image of GCaMP to a 20 Hz, 2 s stimulation was used for differentiation of boutons versus neurites (see white arrows). **B**) Flowchart showing the sequence of a live imaging experiment. Surface fraction was measured by the MES-NH_4_Cl experiment for the same neuron before and after 3 repeated 10 μM dopamine (DA) perfusions. **C**) Representative traces depicting the pHmScarlet-D2S response to 3 repeated DA perfusion (color coded) at boutons of *Synj1*+/+ and *Synj1*+/− neurons. **D**) Summary of the peak fluorescence (ΔF/F_0_ peak) during DA perfusion in *Synj1*+/+ and *Synj1*+/− neurons. *p = 0.0014, *Synj1*+/− boutons, p = 0.24, paired Student’s *t*-test comparing 1^st^ and 3^rd^ DA perfusion **E**) Representative images from the MES-NH_4_Cl experiment before and after DA perfusion. Top panels are baseline fluorescence of pHmScarlet-D2S, and bottom panels are ΔF images of the NH_4_Cl response, which reveal intracellular localization of the pHmScarlet-D2S (arrowheads). **F-I**) Representative traces of surface fraction measurement for boutons (**F, G**) and neurites (**H, I**) of *Synj1*+/+ and *Synj1*+/− neurons. (**J-K**) Summary of pHmScarlet-D2S surface fraction reduction after repeated treatment of DA for boutons (**J**) and neurite (**K**) of *Synj1*+/+ and *Synj1*+/− neurons (*Synj1*+/+ bouton: n=8, *Synj1*+/+ neurite: n=8, *Synj1*+/− bouton: n=9, *Synj1*+/− neurite: n=10). **p<0.01, ***p<0.001, paired Student’s *t*-test. (**L-M**) Comparison for the loss of surface fraction between *Synj1*+/+ and *Synj1*+/− neurons at boutons (**L**) and neurites (**M**). *p= 0.020, two sample Student’s *t-*test.

To further assess any potential trafficking defects of D2S in *Synj1*+/− neurons, we sought to expose these neurons to an endogenous ligand, dopamine. Ligand-induced D2 receptor trafficking has been reported at soma^62–66,79–82^ but was poorly understood in the axons. A previous study of μ-opioid receptors suggested local presynaptic recycling in conjunction with lateral surface diffusion to maintain its surface distribution^45^. To capture differences in D2S trafficking at release sites versus axonal shafts, we co-expressed cultured *Synj1*+/+ and *Synj1*+/− neurons with GCaMP6f and pHmS-D2S (Fig. 5A). Active boutons (presynaptic terminals) were defined by GCaMP hotspots in response to a brief high-frequency stimulation (20 Hz, 2 sec) and the remaining areas were denoted as axons (axonal shafts). We then performed a surface fraction measurement, followed by a 3-time repeated dopamine (10 μM) treatment (3 min each with 3–5 min interval), and ended with a second surface fraction measurement (Fig. 5B). Surface fractions and timelapse pHmS-D2S dynamics were recorded for boutons and axons separately. In nearly all boutons and axons, we observed a dopamine-induced pHmS-D2S fluorescence reduction, indicating a loss of surface D2S (Fig. 5C–D), consistent with the increased intracellular D2S revealed by the NH_4_Cl perfusion (Fig. 5E). The fluorescence reduction began with a 20–30 sec delay following the start of perfusion and partial recovery was observed immediately following dopamine removal. Interestingly, at boutons of *Synj1*+/+ neurons, the dopamine triggered peak fluorescence exhibited a progressive decline, suggesting less internalization over time. This adaptive change was not observed in either boutons or neurites of *Synj1*+/− neurons. To determine whether the lack of adaptive trafficking led to greater internalization of D2S in *Synj1*+/− boutons following repeated dopamine exposure, we examined surface fractions of *Synj1*+/+ and *Synj1*+/− neurons at both axonal compartments. Indeed, we found a significantly more robust loss of surface D2S in boutons of *Synj1*+/− neurons (Fig. 5F, G, J, L), whereas the change in neurites was not different (Fig. 5H, I, K, M). Together, these results suggest that despite the lack of difference at basal conditions, *Synj1*+/− release sites are more prone to losing surface D2S following repeated exposure to dopamine.

### Synj1 and VPS35 share a common pathway to regulate D2S surface expression in axons.

**Fig. 6:**
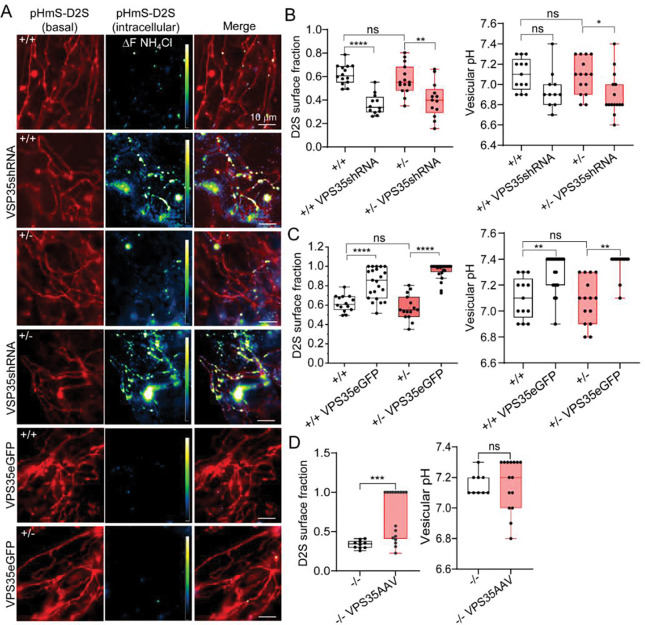
Synj1 and VPS35 function in the same pathway to regulate D2S trafficking and presynaptic surface availability. **A)** Representative confocal images of live neuronal axons showing basal pHmS-D2S fluorescence at axons, intracellular pHmS-D2S revealed by NH_4_Cl, and their merged image. **B-C)** Summary of the measured axonal D2S surface fraction (left) and vesicular pH (right) for *Synj1*+/+ (n=14), *Synj1*+/− (n=15) midbrain neurons expressing pHmScarlet-D2S, pHmScarlet-D2S with BFP-VPS35 shRNA or pHmScarlet-D2S with VPS35-eGFP. Data from 3 batches of cocultures. *p<0.05, **p<0.01, ****p<0.0001 ns = non-significant, two-way ANOVA with Turkey’s multiple comparison. **D)** Summary of the axonal D2S surface fraction (left) and vesicular pH (right) for *Synj1*^*DA*^ cKO dopamine neurons (*−/−,* n=10) and those expressing AAV2/5-DIO-VPS35 (*−/−* VPS35AAV, n=17). ***p<0.001, Mann-Whitney test.

The greater dopamine-induced loss of D2S at *Synj1*+/− boutons could be due to enhanced internalization or reduced endosomal surface delivery. Endosomal recycling pathways are known to be executed by the VPS35-associated retromer complex^43,44,83^. Although the role of VPS35 in D2 receptor recycling has not been reported, previous studies showed that the dopamine transporter is a substrate of VPS35^46,47,50,51^. We thus hypothesized that VPS35 may contribute to D2S endocytic sorting and surface recycling at presynaptic sites. To test this, we modulated VPS35 levels in cultured midbrain neurons by shRNA-mediated knockdown or VPS35 wildtype protein overexpression and examined their impact on surface D2S levels using the pHmS-D2S reporter. VPS35 knockdown reduced D2S surface fraction in *Synj1*+/+ neuronal axons to ~30% (Fig. 6A, B, Supplemental Fig. 3A–C), comparable to what was observed in the *Synj1*^*DA*^ cKO dopamine neurons (Fig. 4). Interestingly, VPS35 knockdown in *Synj1*+/− neurons did not lead to additional exacerbation in the surface D2S level (Fig. 6B), suggesting Synj1 and VPS35 work in a common pathway for D2S recycling. Conversely, VPS35 overexpression significantly increased D2S surface levels in both *Synj1*+/+ and *Synj1*+/− neurons with a commensurate increase in D2S vesicular pH (Fig. 6A, C, Supplemental Fig. 3D). To further investigate this potential Synj1-VPS35 signaling axis in surface D2S regulation, we transduced the *Synj1*^*DA*^ cKO dopamine neurons with a *Cre*-dependent AAV2/5-hSynapsin promotor-DIO-VPS35 with AAV9-hSynapsin promotor-DIO-pHmS-D2S. Overexpressing VPS35 was able to restore the axonal surface D2S from 35% to 70% in *Synj1*^*DA*^ cKO neurons (Fig. 6D, Supplemental Fig. 3E–F), comparable to the control level (Fig. 4C). Thus, increasing VPS35 level is sufficient to override Synj1 deficiency to enhance D2S surface availability.

**Fig. 7:**
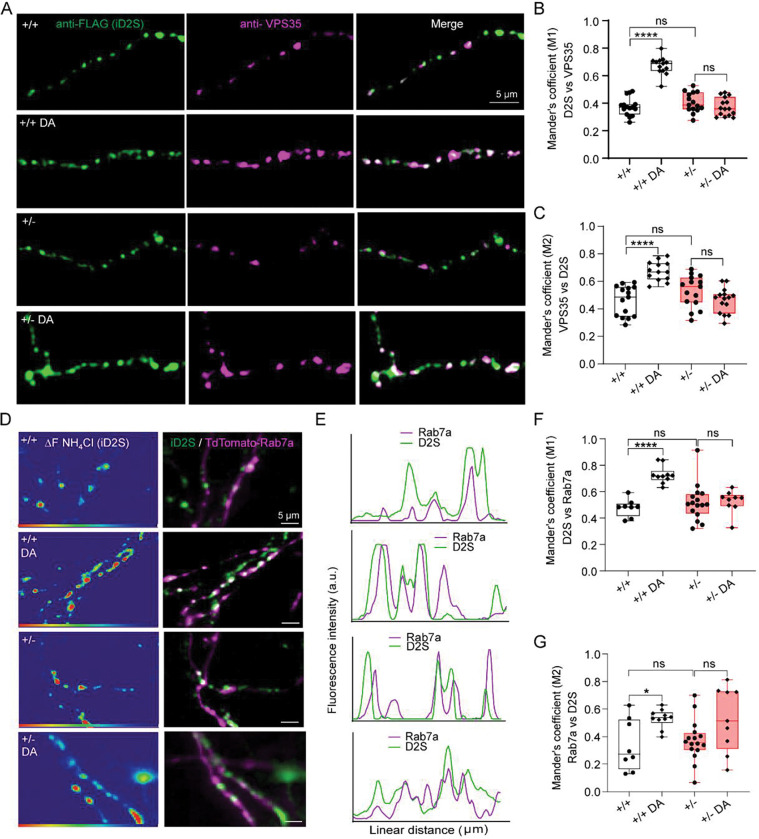
Synj1 is required for axonal recruitment of VPS35 and Rab7a to intracellular D2S containing endosomes. **A)** Ventral midbrain *Synj1*+/+ and *Synj1*+/− neurons expressing FLAG-D2S treated repeatedly with vehicle or 10 μM dopamine (DA), then immunolabeled for anti-VPS35 and anti-FLAG after blocking surface FLAG. Representative confocal images of neuronal axons showing colocalization of VPS35 (magenta) and intra-axonal FLAG-D2S (iD2S, green) in all conditions. **B-C)** Summary of Mander’s coefficient analysis showing colocalization of iD2S with VPS35 **(B)** and VPS35 with iD2S **(C)** in *Synj1*+/+ and *Synj1*+/− neuronal axons after 3 repeated treatments with vehicle or 10 μM DA. **D)** Ventral midbrain *Synj1*+/+ and *Synj1*+/− neurons co-expressing pHluorin-D2S and TdTomato-Rab7a treated repeatedly with vehicle or 10 μM DA, then undergo live perfusion of NH_4_Cl. Representative live axonal images showing ΔF of NH_4_Cl perfusion that reveals intracellular pHluorin-D2S (iD2S) and TdTomato-Rab7a (magenta). **E)** Line intensity profiles showing the colocalization of Rab7a (magenta) with iD2S (green) puncta. **F-G)** Summary of Mander’s coefficient analysis showing colocalization of iD2S with Rab7a **(F)** and Rab7a with iD2S **(G)** in *Synj1*+/+ and *Synj1*+/− neuronal axons. Each data point represents analysis of axons within one field of view. Data from 2 batches of cultures. *p<0.05, **p<0.01, ****p<0.0001, ns = non-significant, two-way ANOVA with Turkey’s multiple comparison.

We next sought to examine whether Synj1 helps recruit VPS35 to its endosomal cargo. In wildtype neurons expressing FLAG-D2S, we observed a 40% colocalization of the intracellular FLAG-D2S (immunolabeled after blocking surface D2S with an antibody) with endogenous VPS35 at baseline (Fig. 7A–C). Notably, a 3-time repeated dopamine (10 μM) treatment induced a substantial increase in their colocalization coefficients (both M1 and M2 using Mander’s coefficient analysis, Fig. 7B–C, and Pearson’s correlation coefficient in Supplemental Fig. 4A). In contrast, the same dopamine treatment in *Synj1*+/− neurons failed to induce an increase in their colocalization (Fig. 7A–D, Supplemental Fig. 4A), suggesting that Synj1 is required for dopamine-induced VPS35 recruitment to endosomal D2S in neuronal axons.

The colocalization study indicated mutual enrichment of D2S and VPS35 within shared endosome compartments. To further define the endosomal compartment in this sorting step, we next assessed colocalization of D2S with Rab7a, which is known to stabilize VPS35 on the endosomal membrane during retromer assisted cargo recruitment^84–86^. In *Synj1*+/+ and *Synj1*+/− neurons co-expressing pHluorin-D2S and TdTomato-Rab7a, we analyzed the colocalization of Rab7a with intracellular D2S in live neurons by perfusing NH_4_Cl. Consistent with the finding in the VPS35 study, repeated dopamine treatment significantly increased D2S-Rab7a colocalization in *Synj1*+/+ axons (in both M1 and M2), however, this effect was not observed in *Synj1*+/− axons (Fig. 7E–H, Supplemental Fig. 4B–C). Together, these data suggest a Synj1-dependent step upstream of VPS35 that is essential for ligand-induced recruitment of VPS35- and Rab7a to D2S-containing endosomes.

### Correlated Synj1-VPS35 expression level and localization in dopaminergic axons.

**Fig. 8:**
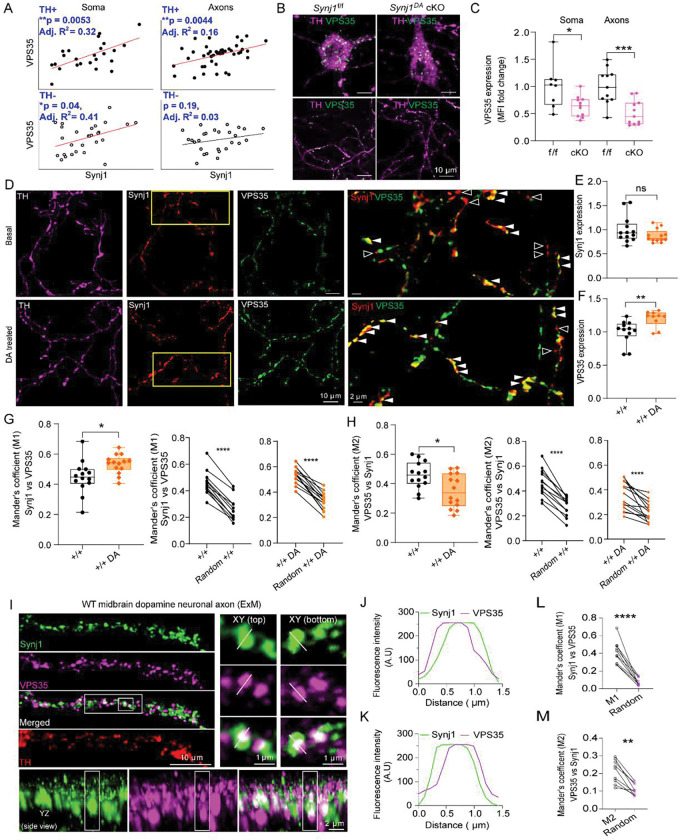
Synj1 and VPS35 exhibit coregulated expression and localization in dopamine neurons. **A)** Scatter plots and their linear fit of Synj1 and VPS35 immunofluorescence at soma and axons. Each symbol represents a soma or an axon by line selection in ImageJ. TH+ soma n=20, TH- soma n=28, TH*+* axons n=43, TH- axons n=28. Data from 3 batches of cultures. p from linear fit model. **B)** Representative confocal images of neurons showing VPS35 (green) expression in TH+ (Magenta) soma and axons in *Synj1*^f/f^ and *Synj1*^DA^ cKO dopamine neurons. **C)** Quantification of VPS35 expression in TH+ soma and axons. *p=0.018 for soma and ***p=0.0007 for axons, Student’s *t-*test. **D)** Representative confocal images of neuronal axons at baseline and after 3 repeated treatments of 10 μM dopamine (DA) immunolabeled with Synj1 (red) and VPS35 (green) and TH (magenta). Yellow boxed areas were magnified on the right. Solid arrowheads point to Synj1-positive structures containing VPS35. Open arrowheads point to Synj1-positive structures do not contain VPS35. **E-F)** Quantification of Synj1 (**E**) and VPS35 (**F**) expressions in TH+ axons. **p=0.0064, Student’s *t-*test. **G, H)** Summary of Mander’s coefficient analysis showing the fraction of Synj1 colocalized with VPS35 (**G,** *p=0.016, Student’s *t-*test) and the fraction of VSP35 colocalized with Synj1 (**H,** *p=0.010, Student’s *t-*test) in WT neuronal axons after 3 repeated treatments with vehicle or 10 μM dopamine. Data from 2 batches of cultures. VPS35/Synj1 colocalization with randomized (40 displacements) controls for each image shown in line plots. ****p<0.0001, paired *t-*test. **I**) Representative ExM images of TH+ axons co-immunolabeled for Synj1 (green), VPS35 (magenta), and TH (red). High magnification view of a representative Synj1-VPS35 punctum shown in XY from top and bottom view. Bottom panel: orthogonal YX view of the same punctum showing close axial apposition of Synj1 and VPS35 signal at nanoscale distances. **J, K)** Line-intensity profile across the puncta, XY top (**J**) and XY bottom (**K**). **L, M)** Mander’s overlap coefficient analysis showing (**L**) Synj1/VPS35 (M1) and (**M**) VPS35/Synj1 (M2) colocalization with randomized lateral shifts (20 displacements) controls. **p<0.01 and ****p<0.0001, paired *t-*test. Data from 2 batches of cultures. Each data point represents axonal analysis within a field of view.

Having established that Synj1 is required for dopamine-induced and VPS35-mediated endosomal sorting of D2S, we next asked whether Synj1 and VPS35 cooperate in a broader sense at the presynaptic terminal. To investigate this, we first examined endogenous VPS35 and Synj1 expressions levels in cultured wildtype neurons containing a mixture of both TH+ and TH- cells. We observed positive linear correlations of VPS35 and Synj1 immunofluorescence in TH+ neurons at both soma and axons (Fig. 8A). Interestingly, the correlation was not significant in the axons of TH- midbrain neurons, suggesting a potentially tighter functional cooperation of the two in dopaminergic axons. We next examined VPS35 expression levels in midbrain dopamine neurons from littermate *Synj1*^*flox/flox*^ and *Synj1*^*DA*^ cKO cultures. Immunofluorescence analysis revealed a significant reduction in VPS35 expression in *Synj1*^*DA*^ cKO dopamine neurons, both in the soma and axons (Fig. 8B, C). These findings suggest that Synj1 is critical not only for endosomal sorting of D2S but also for maintaining sufficient VPS35 levels in dopamine neurons.

To determine whether Synj1 and VPS35 levels and localizations are dynamically regulated by dopamine, we performed the dopamine perfusion experiment like before. In wildtype dopamine neurons, a 3-time repeated dopamine (10 μM) exposure did not affect the Synj1 level but resulted in a significant increase in the endogenous VPS35 level in the axons (Fig. 8D–F). Given the short duration of dopamine treatment, this increase likely reflects mobilization or redistribution of existing VPS35 into axonal compartments. To assess how dopamine affects the spatial localization of Synj1 and VPS35, we quantified their colocalization in axons using Mander’s coefficients. Under basal conditions, ~45% of the Synj1 immunofluorescence overlapped with VPS35 (M1), and ~46% of that from VPS35 overlapped with Synj1 (M2), indicating a partial colocalization in resting neurons. Following repeated dopamine treatment, the proportion of Synj1-positive compartments containing VPS35 increased by ~10% (Fig. 8H), suggesting recruitment. Interestingly, the proportion of overall VPS35 that localized to Synj1-positive compartments decreased by ~10 % (Fig. 8I), suggesting that dopamine-induced increase in axonal VPS35 is also utilized elsewhere in addition to its cooperation with Synj1. To determine whether the immunolabeling of Synj1 and VPS35 are true colocalization rather than random coincidence, we randomized the VPS35 signal in each condition (see methods for details) and recalculated Mander’s overlap coefficient (M1 and M2). All randomized colocalization measurements of Synj1 and VPS35 were significantly lower, supporting biological colocalization of Synj1 and VPS35 in dopaminergic axons.

To further determine the nanoscale organization of Synj1 and VPS35 molecules within dopaminergic axons, we performed expansion microscopy^87,88^ (ExM, 4.2×) that exhibit lateral resolution of 50–70 nm. This analysis revealed that VPS35 puncta were frequently embedded within the larger patches of Synj1-postive structures, forming discrete nanoscale assemblies along TH+ axons (Fig. 8I). Line-intensity profiles across individual puncta showed that Synj1 and VPS35 were largely coincident but also exhibited a small, consistent offset at the edges, indicating co-clustering of VPS35 and Synj1-postive compartments (Fig. 8J, K). Consistent with this nanoscale organization, Mander’s colocalization coefficient for Synj1 with VPS35 (M1) was 41% and that for VPS35 with Synj1 was 21%. Importantly, both values were significantly higher than randomized controls (Fig. 8L, M), demonstrating the partially overlapping nanoscale organization of the two molecules. Together, these studies provide evidence that dopamine promotes VPS35 recruitment into Synj1-enriched domains within neuronal axons.

## Discussion

The molecular regulation of presynaptic sorting remains poorly understood. Here we demonstrate that Synj1, a presynaptically enriched molecule well-known for its role in synaptic vesicle recycling^89–92^, colocalizes and functionally cooperates with the retromer component VPS35 to regulate dopaminergic signaling. Our data further suggest that Synj1 acts upstream of VPS35, regulating its expression and axonal localization, particularly promoting its recruitment to presynaptic terminals following dopamine exposure. Loss of Synj1 in neurons impairs the recruitment of VPS35 and Rab7a to the endosomal cargo D2S. In addition to marking late endosomes, Rab7a facilitates VPS35 stabilization and membrane tethering, steps essential for cargo recognition and sorting. Therefore, disrupted recruitment results in accumulation of D2S in endosomal compartments. These endosomal trafficking defects were observed in *Synj1*+/− neurons following repeated dopamine exposure and in *Synj1−/−* dopamine neurons at baseline and can be rescued by increasing VPS35. Thus, the Synj1-VPS35 functional cooperation is likely essential for regulating D2-dependent dopamine signaling. This is evidenced by impaired responses to a D2-like antagonist at dopaminergic boutons *in vitro*, and to a D2-like agonist *in vivo* during locomotor behavior. Together, our findings identify a novel cooperation between a synaptic vesicle endocytic protein and an endosomal sorting machinery in regulating dopamine signaling. Disruption of this pathway may impair the ability of dopaminergic terminals to adapt to synaptic demand, potentially contributing to early dysfunction and selective vulnerability of dopaminergic neurons.

Emerging evidence has suggested broader roles of Synj1 in membrane trafficking, such as autophagy^28,93,94^, lysosome homeostasis^93,95^, and regulation of surface cargos^33,35,78^. Our findings provide mechanistic insight into its role in surface cargo delivery by showing that Synj1 colocalizes and functionally cooperates with the retromer component VPS35. We showed that Synj1 controls VPS35 function via at least two distinct mechanisms. First, Synj1 is required to maintain VPS35 levels, especially in dopaminergic axons. How Synj1 regulates VPS35 abundance is unclear at present. One possibility is that Synj1 loss alters endosomal phosphoinositide composition and/or impairs Rab7a-dependent stabilization^96^, thereby destabilizing VPS35 and leading to its mislocalization and degradation. The degradation of VPS35 may be further enhanced by elevated lysosomal proteolytic activity, as previously reported in Synj1-deficient neurons^95^, suggesting a conserved role for inositol phosphatases in regulating endolysosomal degradation capacity. Second, Synj1 is required to support VPS35 recruitment to endosomal cargos. We found that Synj1 deficiency prevents ligand-induced recruitment of VPS35 to D2S-positive endosomes. This function of Synj1 is potentially mediated by its role in endosomal lipid remodeling, which allows interaction with the PX domain on sorting nexin (SNX) family proteins^97^, thereby facilitating VPS35 recruitment. Thus, Synj1 integrates lipid signaling with retromer-dependent endosomal cargo sorting - a process likely essential for maintaining surface cargo availability. Interestingly, our data showed that increasing VPS35 levels was sufficient to overcome the endosomal lipid defect in *Synj1*-null neurons to deliver axonal surface D2S. This data suggests that either additional lipid phosphatases were upregulated to compensate for the loss of Synj1 in remodeling endosomal lipids, or VPS35 recruitment is the rate-limiting step in surface cargo delivery.

The cooperation of Synj1 and VPS35 may affect the presynaptic sorting of multiple surface cargos, which awaits further investigation. In this study, we focused on analyzing a dopamine neuron specific presynaptic surface cargo – the dopamine D2 autoreceptor. We showed that D2S function is impaired in *Synj1*+/− neurons and mice. Although we do not have direct evidence for D2S desensitization, the increased surface PI(4,5)P_2_ level in *Synj1*+/− dopamine neurons^71^ will likely lead to enhanced activation^98,99^ followed by desensitization of the D2S receptor. Thus, receptor desensitization may be the predominant effect in *Synj1*+/− neurons. Repeated dopamine exposure consistently triggers D2S internalization, which requires endosomal sorting to reestablish its surface levels to maintain dopamine signaling at active synapses. We show that Synj1 deficient synapses cannot meet this demand. This is supported by *in vitro* and *in vivo* evidence suggesting a lack of pharmacological responses of endogenous receptors as well as the altered fluorescence response of pHmS-D2S in Synj1 deficient conditions. It is worth noting that overexpression of the pHmS-D2S may lead to altered trafficking and distribution of endogenous D2S. The localization of pHmS-D2S should not be overinterpreted and whether the dopamine-induced pHmS-D2S dynamics represents the *bona fide* D2S response to dopamine awaits future validation. Previous studies showed that D2S functions upstream of DAT to regulate its localization, conformation, as well as the VPS35-dependent surface delivery^48,100^. In *Synj1*+/− neurons, we have found higher extracellular tonic dopamine levels^35^ (nanomolar range^101^). Thus, it is possible that desensitized D2S in *Synj1*+/− neurons contributes to the loss of surface DAT at baseline. In healthy neurons, an increase in desensitized D2S likely triggers intracellular signaling to promote endosomal sorting for surface delivery of new D2S. This process, however, is impaired in Synj1 deficient neurons, especially during repeated dopamine exposure, or in physiological conditions during repeated high-frequency neuronal activity mimicking phasic release (produces dopamine in the millimolar range^101^). Our results thus suggest that receptor desensitization is perhaps not sufficient to drive internalization. Rather, the trigger for D2S internalization could be from the endosomes. D2S is only internalized following insertion of functional receptors. Therefore, maintaining endosomal functions may be key to the integrity of dopaminergic signaling.

Both Synj1 and VPS35 are linked to PD, their convergent signaling in presynaptic endosomal sorting and in disrupting dopamine signaling may represent an essential molecular pathway contributing to early pathogenesis. Dysfunctional dopamine release, especially changes in DAT and D2R expression and functions are found in several other genetic models of PD^102–107^, including the VPS35 PD mutation knockin mice^46,50,51,108^, many of which do not exhibit neurodegeneration or motor symptoms. *Synj1*+/− male mice exhibit impaired locomotor coordination on the accelerated Rotarod at 12 months^71^, however, as early as 3 months, these mice already showed impaired behavioral responses to agonists of DAT^35^ and D2 receptor (this study). Intriguingly, female *Synj1*+/− mice do not exhibit defects in either DAT or D2 receptor responses, suggesting a male-specific vulnerability to Synj1 deficiency in maintaining dopaminergic signaling *in vivo*. Although this sex-specific effect is not currently understood, it could be due to higher levels of phosphorylated inositol lipids in male compared to female brains^109,110^ that require higher Synj1 phosphatase activities for local trafficking. Altered surface availabilities of DAT and D2R are also found in functional brain scans of early-stage and prodromal PD patients and consistently observed in VPS35 related PD models. Thus, the Synj1-VPS35 signaling in presynaptic endosomal sorting, especially in regulating D2S availability and function likely represent a key pathway in triggering dopaminergic vulnerability in early PD. A recent study suggested the involvement of Synj1 in endosomal sorting of transferrin receptors^33^. Given the essential role of transferrin receptors in transporting iron for lysosomal and mitochondrial function, and the well-established contribution of iron dysregulation to neurodegenerative disorders, it remains to be investigated whether the Synj1-VPS35 signaling is at play for iron homeostasis and iron-associated oxidative stress. VPS35-based therapy has been proposed and shown to be effective in various models of neurodegenerative disorders including PD. Whether it can be used for correcting the surface proteome of dopaminergic neuron during PD pathogenesis awaits further investigation.

## Materials and Methods

### Ethical approval

All animal studies were conducted in accordance with the National Institutes of Health (NIH) and with protocols (PROTO201800183) approved by the Institutional Animal Care and Use committee (IACUC) of Rutgers University.

### Animals

Mice were housed under normal 12 h day/night cycle in a pathogen-free barrier facility at the Rutgers Robert Wood Johnson Medical School Research Tower vivarium. The *Synj1*+/− mouse was originally gifted by the Pietro De Camili laboratory at Yale University. The *C57BL/6J* mice were purchased from the Jackson laboratory. *C57BL/6J* mice were crossed with *Synj1*+/− mice to generate littermate mice for midbrain culture and for behavioral studies. The number of mice used in each behaviors test is included in figures and figure legend. The Synj1 condition ready allele (Tm1c) was generated by MRC Harwell (UK) with *loxP* sites flanking the critical exon 4. *Synj1*^*flox/flox*^ mice were identified using 5arm-WTF and Crit-WTR primers, which produces a single band that migrates at ~550 bp. *Synj1*^*flox/flox*^ mice was crossed with heterozygous *DAT*^*IRESCre*^ (*DAT-Cre*) mice (Jackson Laboratory, stock# 06660) for two generations to obtain the *DAT-Cre(+/−);Synj1*^*f/f*^ or *Synj1*^*DA*^ cKO mice. These mice were viable and fertile and the conditional deletion of *Synj1* in ventral midbrain dopamine neurons has been confirmed. The *Synj1*^*DA*^ cKO mice were crossed with *Synj1*^*flox/flox*^ mice to generate 50% *Synj1*^*DA*^ cKO pups following mendelian inheritance. The *DAT-cre* cultures were prepared separately by crossing *DAT-Cre(+/−)* with *C57BL/6J* mice. This breeding allows sufficient numbers of pups born in a litter with the desired genotype for pooled ventral midbrain culture containing pups of both sexes.

### Locomotor activity test

Mice were brought to the behavioral room 1 hour before the test for habituation. Two cohorts of mice containing both male and females were split to be injected intraperitoneally with either saline or quinpirole (0.5 mg/kg, Tocris, Cat #1061), freshly prepared in sterile saline. Immediately following the injection, animals were placed into the center of a clean 19 × 19-inch cage with a Versamax monitor system (Accuscan) and allowed to explore freely for 45 min. Animal movement was recorded by beam breaks built into the system, which quantified the total distance traveled over the testing period. Chambers were thoroughly cleaned between animals. All behavioral testing was conducted during daytime.

### Antibodies

The following primary antibodies were used in this study: polyclonal anti-guinea pig synapsin I/II (Synaptic System, Cat. #106004, 1:500), polyclonal anti-goat VPS35 (Novus Biologicals, Cat. #NB100–1397; 1:500), rabbit anti-TH (Novus Biologicals, Cat. #NB300–109, 1:1000), mouse anti-TH (Sigma, Cat. #T2928, 1:1000), polyclonal chicken anti-TH (Sigma-Aldrich, Cat. #AB9702), polyclonal anti-rabbit Synj1 (Cat. #NBP1–87842; 1:500), monoclonal mouse anti-FLAG (Sigma-Aldrich, Cat. #F1804; 1:500), polyclonal rabbit anti-Flag (Proteintech, Cat. #20543–1-AP; 1:500). Secondary antibodies: Donkey anti-mouse Alexa Fluor 488 (Invitrogen, Cat. #A-21202; 1:1000), donkey anti-rabbit Alexa Fluor 555 (Invitrogen, Cat. #A-31572; 1:1000), donkey anti-chicken Alexa fluor 488 (Invitrogen, Cat. #A78948; 1:1000), donkey anti-goat Alexa Fluor 647 (Invitrogen, Cat. #A-21447; 1:1000), Donkey Anti-Goat IgG ATTO647N (Hypermol, Cat. #2910; 1:250).

### Solutions and buffers

All solutions were prepared using analytical-grade reagents (Sigma-Aldrich, unless otherwise noted) and adjusted to the appropriate pH using NaOH or HCl.

Tyrode’s solution (pH 7.40): 30 mM Glucose, 25 mM HEPES, 2.5 mM KCl, 119 mM NaCl, 10 μM 6-cyano-7- nitroquinoxaline-2,3-dione (CNQX), 2 mM MgCl_2_, 2 mM CaCl_2_ and 50 μM D,L-AP-5. The MES buffer: (pH 5.50): 25 mM MES, 30 mM D-glucose, 70 mM NaCl, 2 mM MgCl_2_, 2.5 mM KCl, 2 mM CaCl_2_, 50 μM AP-5, 10 μM CNQX, and buffered to pH 5.50. NH_4_Cl buffer: (pH 7.40): 50 mM NH4Cl, 30 mM Glucose, 2.5 mM KCl, 2 mM MgCl_2_, 70 mM NaCl, 2 mM CaCl_2_, 25 mM HEPES, 50 μM AP-5 and 10 μM CNQX.

### Constructs and virus

The pCAGP-FLAG-pHmScarlet/pHluorin-hD2S were engineered using a previously published pCAGP-hDAT-pHluorin backbone^78^. The pHmScarlet cDNA was amplified from VAMP2-pHmScarlet, Addgene #166890. The following linkers were added on the 5’ and 3’ of the pHmScarlet, respectively: TGSTSGGSGGTGG and SGGTGGSGGTGGSGGTG. The linker-pHmScarlet-linker sequence was subcloned into pcDNA-FLAG-hD2S-L-Venus (Addgene, Cat. #19966) via an inserted Age-I restriction site immediately following a signal peptide (MKTIIALSYIFCLVFA) and a FLAG sequence. The open reading frame (ORF) containing signal peptide-FLAG-linker-pHmScarlet-linker-D2S was then subcloned into the pCAG backbone vector replacing the hDAT-pHluorin. The ORF was validated by sequencing before use. The same ORF was synthesized into a pAAV9-hSYNp-DIO vector by commercial service, VectorBuilder and packaged into high titer (>10e13 GC/mL) AAV9 particles for transduction of *DAT-cre* and *Synj1*^*DA*^ cKO cultures. The pDEST-eGFP-C1-VPS35 was purchased from addgene (Cat. #163622). The AAV2/5-hSYNp-DIO-BFP-hVPS35-tWPA was synthesized and packaged at high titer (>10e12 GC/mL) by OBiO Inc. pRP-TagBFP2-U6>mVPS35 shRNA was designed and synthesized by VectorBuilder using the following targeting sequence: AGCTTAACCTTGAACATATTG.

### Neuronal culture, transfection and AAV transduction

Primary ventral midbrains (MB) cultures were prepared from postnatal day 0–1 (P0-P1) *Synj1*+/+, *Synj1*+/−, *DAT-Cre+/−* and *Synj1*^*DA*^ cKO mouse pups as previously described^35,71,77^. The genotype of each pup was determined by PCR and at least 2~3 pups in the same genotype were pooled to prepare the culture. As described before^77^, MB tissue containing the substantia nigra (SN) and ventral tegmental area (VTA) was dissected and digested using papain (Worthington, Cat. #LK003178) in a 34–37°C constant oxygenated water bath with gentle stirring. Dissociated neurons were then seeded at a density of 3 × 10^4^ cells per 0.28 cm^2^ area within cloning cylinders placed on poly-L-ornithine-coated coverslips (Sigma, Cat. # P3655). Cells were maintained in Neurobasal-A medium (Gibco, Cat. #12349015) supplemented with 10 ng/mL glial cell-derived neurotrophic factor (GDNF; EMD Millipore, Cat. # GF030).

At DIV 5–7, neurons were transfected with the indicated plasmid DNA using Lipofectamine 2000 (Thermo Fisher, Cat. #11668019) as per the manufacturer’s instructions with minor modifications. After 45 min of incubation at 37°C, the transfection mixture was washed off with 1x MEM, and cultures were returned to fresh medium containing GDNF and cytosine β-arabinofuranoside (Ara-C) to suppress glial proliferation. Imaging experiments were conducted between DIV 13 and DIV 17.

For viral transduction, 1 μL of the high titer AAV9 or AAV2/5 particles were added directly onto the neuronal culture grown within the cylinder on DIV 3. After 3 days of incubation, a full media change was conducted, and cylinders were removed until imaging experiments on and after DIV 13. All procedures were in accordance with institutional guidelines on handling hazardous materials and waste management.

### Sniffer cell culture and co-culture with MB neurons

GRAB_DA2m_-expressing sniffer cells^75^ were maintained in DMEM (Thermo Fisher Cat. #11965092) medium supplemented with 10 % fetal bovine serum (Atlanta Biologicals, Cat. #S11550H), 1 % Pen/Step solution (Thermo Fisher, Cat. #15140122), 15 μg/mL Blasticidin (Millipore Sigma, Cat. #15205), and 200 μg/mL Hygromycin (Millipore Sigma, Cat. #H3274). Cells were cultured a humidified incubator at 37°C with 5 % CO_2_.

For co-culture experiments, sniffer cells were seeded at a density of 20,000 cells per 8 × 8 cloning cylinder onto DIV 13 midbrain neuronal cultures in neuronal medium. After overnight incubation, doxycycline (1 μg/mL) was added to induce GRAB_DA2m_ expression for 24 hours. Imaging was performed at DIV 15. Field electrical stimulation was applied using a custom-built stimulation chamber with two platinum electrodes. 1 msec square pulses were generated and delivered by an A310 Accupulser and A385 stimulus isolator (World Precision Instruments) to evoke action potential. Dopaminergic axons were determined blindly based on sniffer cell responses and verified *post hoc* by immunofluorescence against TH and Synapsin I/II.

### Live-cell imaging of pHmscarlet-D2S in cultured neurons

Live imaging of pHm-D2S-expressing midbrain neurons was performed in custom-designed laminar-flow chamber equipped with continuous gravity perfusion, maintained at ~ 30°C. Cells were initially perfused with Tyrode’s solution at a rate of 0.2–0.4 mL/min. For pH-sensitive fluorescence measurements, MES buffer (pH 5.50) was perfused to quench surface-localized fluorescence, and NH_4_Cl buffer (pH 7.40) was applied to neutralize intracellular acidic compartments and reveal the total D2S fluorescence. Imaging was performed using our Nikon Ti2 wide-field inverted microscope equipped with 60x oil-immersion objective and a back-illuminated EM-CCD camera (Andor iXon + DU-897). Images were acquired at 1 frame per second using NIS-element software. Neuronal positions were saved according to a reference point for *post hoc* immunofluorescence analysis.

### Immunofluorescence

Primary midbrain neurons were seeded on poly-L-ornithine-coated coverslips as described above. For experiments involving dopamine treatment, neurons were perfused with freshly prepared 10 μM dopamine in Tyrode’s solution for 3-min pulses, each followed by a 3-min wash with Tyrodes’ solution. Immediately after the final dopamine perfusion, cells were fixed in pre-warmed 4 % paraformaldehyde (PFA) for 10 min at room temperature (RT) followed by PBS washes. Cells were permeabilized with 0.2 % Triton X-100 diluted in PBS for 15 min at RT, washed three times in PBS, and blocked in 5 % bovine serum albumin (BSA) for 45 min. Following blocking, cells were incubated overnight at 4°C with primary antibodies against VPS35, synj1, and tyrosine hydroxylase (TH), diluted in blocking buffer. The next day, cells were washed in PBS and incubated with fluorophore-conjugated secondary antibodies for 1 hour at RT. After washing, coverslips were mounted using Clear-Mount solution (Invitrogen, Cat. #00–8-10) and stored in the dark until imaging.

For surface and intracellular FLAG-D2S immunostaining, neurons transfected with FLAG-D2S were fixed with 4% PFA for 10 min at RT, followed by washed three times with PBS. Surface FLAG staining was performed by blocking the cells in 5 % BSA for 45 min at RT, then incubating with rabbit anti-FLAG primary antibody (1:500) diluted in blocking buffer overnight at 4°C. After washing, cells were incubated with fluorophore-conjugated secondary antibody for 1 hour at RT. Following final washes cells were processed for intracellular labeling. For intracellular FLAG detection, neurons were permeabilized with 0.2 % Triton X-100 diluted in PBS for 15 min, re-blocked in 5 % BSA for 45 min. Cells were then incubated overnight at 4°C with a second mouse anti-FLAG primary antibody (1:500), together with either anti-TH (1:1000), anti-VPS35 (1:500), or Anti-RFP (1:500, for rab7a detection), diluted in blocking solution. After washing with PBS, appropriate fluorophore-conjugated secondary antibodies were applied for 1 h at RT. Finally, cells were washed, and coverslips were mounted onto slides using clear-mount solution. Images were acquired using our confocal microscopy.

### Confocal Imaging

Images for localization analysis were acquired using a Nikon CREST spinning disk confocal microscope. The system includes laser lines at 405 nm, 488 nm, 561 nm, 647 nm, and 701 nm for multi-channel fluorescence imaging. A 100 X oil-immersion objective was used for all acquisition. Laser power, exposure time, and binnings were kept constant within each experimental group. Z-stacks were collected at 0.7 μm step using Nikon Elements software.

### Confocal microscopy image analysis

All image analyses were performed using Fiji (ImageJ). For Synj1 and VPS35 colocalization in axons, maximum intensity projections were generated from confocal z-stacks images. Background subtraction was performed using the rolling ball algorithm (radius = 50). Axonal regions of interest (ROIs) were manually delineated by using TH-positive signal as a guide. Manual intensity thresholds were applied separately to each channel and held constant across conditions. Colocalization analysis was carried out using the BIOP JACoP Plugin to calculate Mander’s overlap coefficients (M1 and M2), measuring the fraction of signal in one channel overlapping with the other within axonal ROIs.

To control for random spatial coincidence, we implemented a lateral shift-based randomization strategy as previously described^111–113^, with slight modifications for axon specific analysis. A custom-written Fiji macro was used to perform 40 independent lateral displacements of the VPS35 channel relative to the fixed Synj1 channel, each involving unique x- and y -axis shifts that preserved signal structure but disrupted spatial alignment. Mander’s overlap coefficients (M1 and M2) were recalculated for each randomized configuration using the JACop, and the average of these 40 values used for comparison. This was then compared to the unshifted (true) Mander’s coefficient obtained from the original, aligned images. For each experimental condition, 14 images were analyzed independently, with each image contributing a signal data point derived from the average for 40 randomized values. Statistical significance between true and randomized colocalization was assessed using a paired, two-tailed t-test.

Expression levels of VPS35 and Synj1 in TH+ axons and soma were quantified using Fiji. Background subtraction was performed using the rolling ball algorithm. TH+ axons were identified by thresholding the TH channel and converting it into a binary mask using the “create selection” function. These same masks were applied to the VPS35 and Synj1 channels, and mean fluorescence intensity was measured within the masked axonal regions. For soma analysis, ROIs were manually outlined using the freehand tool based on TH signal. As a validation step, an alternative approach was used in which ROIs were manually drawn, and background-subtracted mean intensities were calculated by measuring signal from nearby non-axonal regions. Both approaches yielded comparable results. Only values obtained using the rolling ball-corrected analysis were used in the final quantification and statistical comparisons. For representative images shown in figures, the smooth filter in Fiji was applied to minimize pixel noise and improve visualization.

### Expansion microscopy (ExM)

Following immunostaining, samples were processed for expansion according to the published protocol^114^ with minor modifications. Samples were incubated overnight at room temperature in 0.1 mg/ml Acryloyl-X SE (AcX; Invitrogen, Cat. #A20770) prepared by diluting a 10 mg/mL AcX/DMSO stock 1:100 into 1× PBS. The next day, samples were washed two times in 1× PBS for 15 min each.

#### Gelation:

A monomer stock solution (Stock X) was prepared containing 4 M sodium acrylate (Sigma, Cat. #408220), 7M acrylamide (Sigma, Cat. #A9099), 130 mM N, N’-methylenebisacrylamide (Sigma, Cat. #146072), and 5 M sodium chloride in 1× PBS. Immediately before embedding, Stock X was mixed with ddH_2_O, 10% TEMED, and 10% APS at a 47:1:1:1 (V/V) ratio to prepare the gelling solution. Samples were placed in custom gelation chambers, filled with this mixture, and polymerized at 37°C for 1 h.

#### Digestion:

After polymerization, gels were incubated in digestion buffer containing 0.5% Triton X-100, 1 mM EDTA (pH 8), 50 mM Tris-HCl (pH 8), and 800 mM sodium chloride, supplemented with proteinase K (NEB, Cat. #P8107S) at a final concertation of 8 U/mL. Digestion was carried out in dark for 2 h at room temperature.

#### Gel expansion and expansion factor (EF) calculation:

Gels were expanded by three sequential washes in ultrapure water (20 min each) until reaching stable size. To calculate the EF, gels were imaged with a ruler placed adjacent to them before and after expansion. Gel dimensions were measured in Fiji, and EF was calculated as post-expansion gel length divided by the pre-expansion gel lengths. Measurements were taken at multiple positions along each gel and averaged to yield a single EF per gel. Across replicates, the mean linear EF was ~4.2×.

#### Imaging:

Expanded gels were mounted cell-side down on poly-D-lysine-coated coverslips (1 mg/mL, coated overnight, and rinsed in water) within a standard imaging chamber and secured with a custom holder. Ultrapure water was added to fully cover the gel and prevent drying or shrinkage during imaging. Post-expansion imaging was performed on a Nikon CREST spinning-disk confocal microscope equipped with a 100× oil-immersion objective (Plan Apo λ, NA 1.45, n = 1.515) using a fixed pinhole array of 50 μm, equivalent to ~1 Airy unit. Excitation/emission settings were 477/510 nm for Synj1 (Alexa Fluor 488), 546/595 nm for TH (Alexa Fluor 555), and 638/685 nm for VPS35 (Atto 647). Images were acquired at 0.11 μm/ pixel (1200 × 1200) and 0.7 μm Z-steps. Diffraction-limited resolutions were estimated as 0.61 λ / N A (lateral) and 1.4 n λ / NA^2^ (axial), corresponding to ~215/515 nm (XY/Z) at 510 nm, ~250/600 nm at 595, and ~288/691 nm at 685 nm. According to Nyquist sampling criteria, the target sampling is ~0.11 μm (510 nm), ~0.125 μm (595 nm), and ~0.144 μm (685 nm) in XY and ~0.26 μm (510 nm), ~0.30 μm (595 nm) and ~0.35 μm (685 nm) in Z. With a measured 4.2× linear expansion, the effective sampling improves to ~0.026 μm/pixel (XY) and ~0.167 μm (Z), and the effective diffraction-limited lateral resolution (XY/4.3) is ~51 nm (510 nm), ~60 nm (595 nm), and ~69 nm (685 nm).

#### ExM image analysis:

Images were analysed using Fiji (ImageJ) and representative 3D rendering were generated using Imaris (Version 10). Background was subtracted using a rolling ball algorithm (radius = 50). Axonal regions of interest (ROIs) were manually delineated by using TH-positive labelling along with Synj1-positive signal as a guide. Colocalization analysis was performed with the BIOP JACoP Plugin to calculate Mander’s overlap coefficients (M1 and M2). Intensity thresholds were set manually for each channel and then held constant across conditions. To control for random spatial coincidence, we implemented a lateral shift-based randomization strategy as described above. A custom Fiji macro was used to perform a minimum of 20 independent lateral displacements of the VPS35 channel relative to the fixed Synj1 channel, each involving unique x- and y -axis shifts that preserved signal structure but disrupted spatial alignment. Mander’s coefficients were recalculated for each randomized configuration in JACoP, and the average of these 20 randomized values was compared with the unshift (true) coefficient from the original aligned images. For each experimental condition, 9 images were analyzed independently, with each image contributing a signal data point derived from the average for 20 randomized values. Statistical significance between true and randomized colocalization was determined using t-test.

### Statistical analysis and reproducibility

All statistical analyses were performed using OriginLab or GraphPad Prism (v10). Unless specified otherwise, experiments were independently repeated at least two times using neuronal cultures derived from a minimum of two separate litters. Two-tailed Student’s *t-*tests or Mann-Whitney non-parametric tests were used for comparisons between two groups. One-way or two-way ANOVA with appropriate *post hoc* corrections was used for multiple group comparisons. Statistical details including n values, tests, and *p* values are provided in the figure legends. Data are presented as mean ± SEM and p-value < 0.05 was considered statistically significant.

## Supplementary Material

Supplement 1

## Data Availability

All data supporting the findings of this study are presented in the main figures and provided as supplemental materials. Additional datasets or materials are available from the corresponding author upon reasonable request.
