# Supplementary material for: Synaptojanin-1 supports VPS35-dependent trafficking of dopamine D2 autoreceptors at presynaptic terminals
Source: Res Sq. 2025 Oct 15:rs.3.rs-7730224. Preprint. [Version 1] doi: 10.21203/rs.3.rs-7730224/v1 (PMC12633164; doi:10.21203/rs.3.rs-7730224/v1)
Supplement: Supplement 1 [file NIHPPrs7730224v1-supplement-1.pdf]

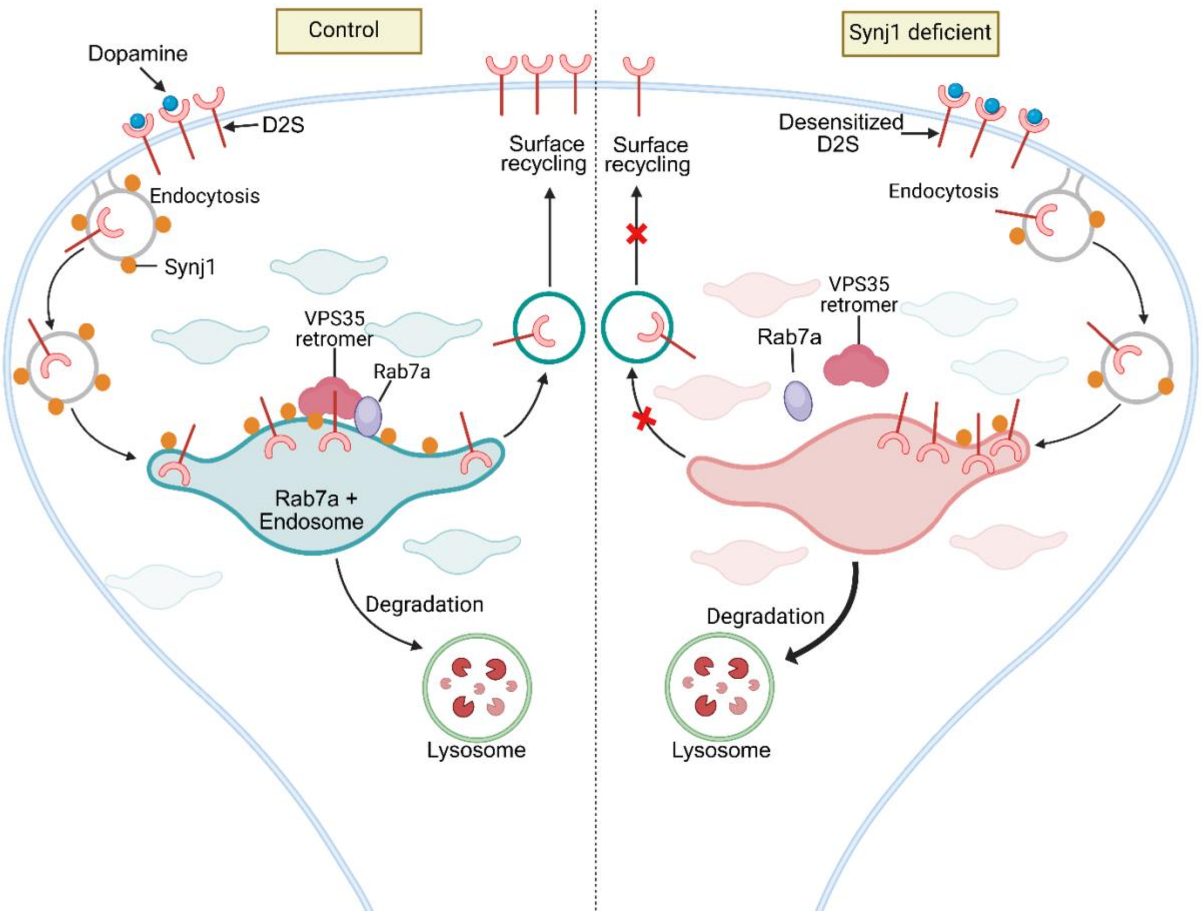

Supplemental Figure 1: Hypothesized model of Synj1-VPS35 cooperation in regulating presynaptic endosomal sorting of the D2S receptor. Surface and intracellular D2S autoreceptors are subject to homeostatic regulation for proper presynaptic release of dopamine. Synj1 remodels endosomal lipids, thereby controlling the recruitment of VPS35 and Rab7a to D2S-containing endosomes. This process is essential for the recycling of D2S back to the surface. In Synj1 deficient conditions, defective lipid remodeling disrupts endosomal identity, reduces VPS35/Rab7a recruitment, thereby limiting retromer-dependent D2S recycling. This recycling defect results in enhanced degradation of intracellular D2S and dysregulated dopamine signaling.

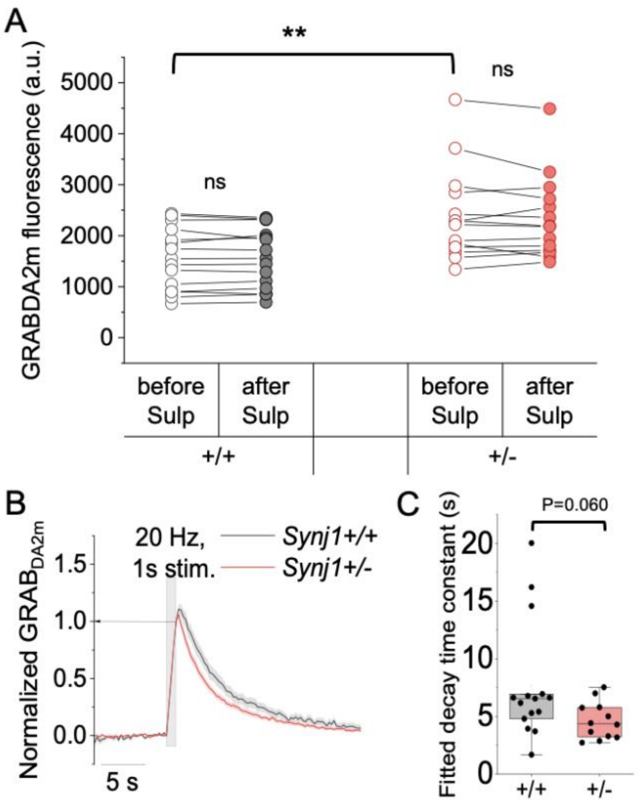

Supplemental Figure 2: Basal GRAB$_{DA2m}$ responses in *Synj1+/+* and *Synj1+/-* cultures. A) Basal GRAB$_{DA2m}$ fluorescence analyzed for all neurons, *Synj1+/+* (n = 18) and *Synj1+/-* (n = 17) before and after a 2-minute sulpiride incubation. ns = not significant from Paired *t*-test. **p<0.01 from two-tailed Student's *t*-test. **B**) All GRAB$_{DA2m}$ responses from *Synj1+/+* (n = 18) and *Synj1+/-* (n = 17) neurons were normalized to their fluorescence at the end of stimulation. Data = mean ± S.E.M. from 5 batches of cocultures. (**C**) Summary of the fitted GRAB$_{DA2m}$ fluorescence decay time constant in all neurons responded positively to sulpiride and *Synj1+/-*, Mann-Whitney test.

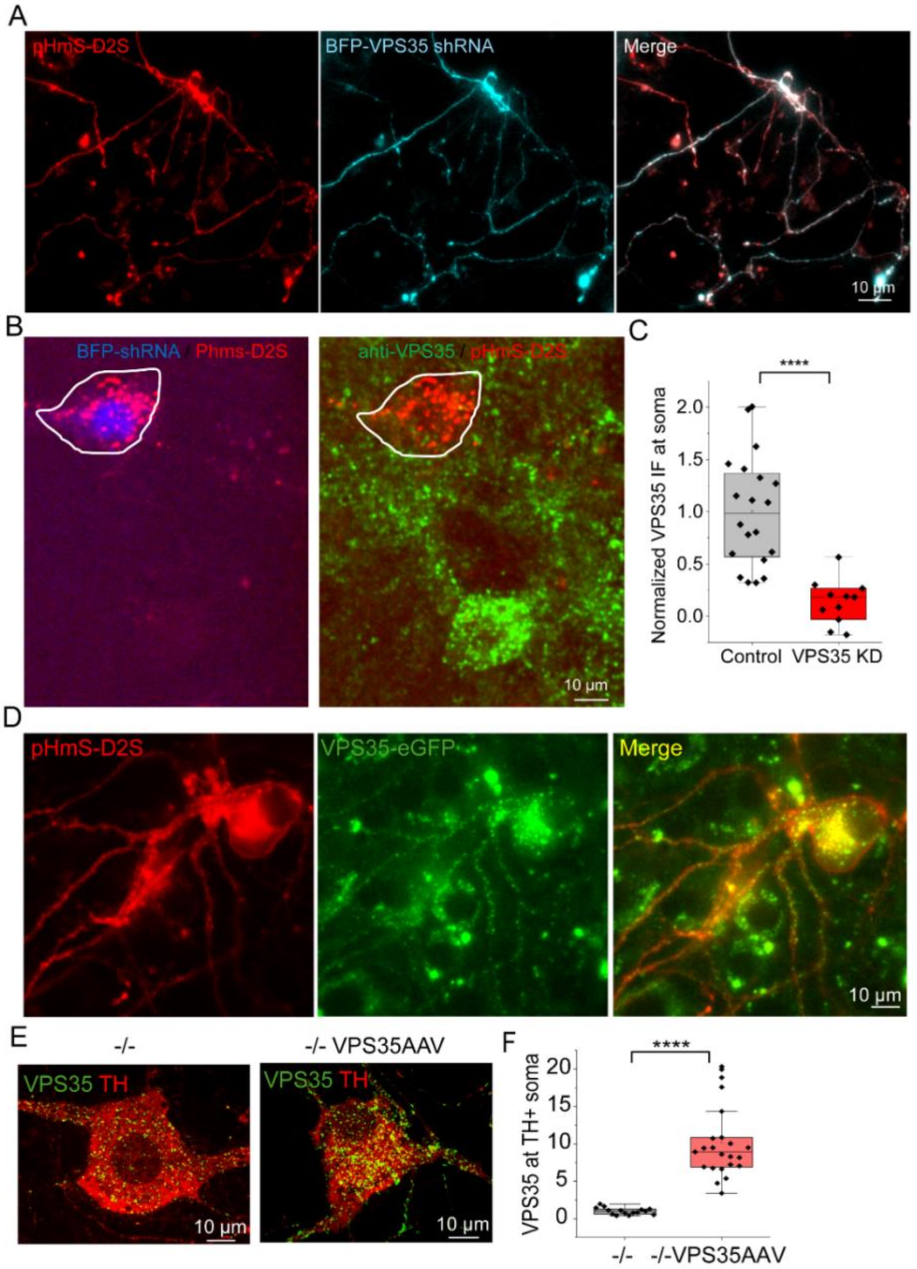

Supplemental Figure 3: Validation of VPS35 knockdown and overexpression in cultured neurons. A) Representative images of ventral midbrain neurons co-expressing pHms-D2S and BFP-VPS35 shRNA. **B)** Representative immunostained images of neurons co-expressing pHms-D2S and BFP-VPS35 shRNA were stained with an anti-VPS35 to measure VPS35 expression. **C)** Measurements of VPS35 expression in soma. **D)** Representative images of ventral midbrain neurons co-expressing pHms-D2S and VPS35 eGFP. **E)** Representative images of a *Synj1*^{DA} cKO dopamine neuron (-/-) soma and a *Synj1*^{DA} cKO dopamine neuron expressing AAV-DIO-VPS35 immunolabeled with anti-VPS35 and anti-TH. **F)** Fold change of VPS35 immunofluorescence at TH+ soma following AAV transduced expression. ****$p<0.0001$, Student's *t*-test.

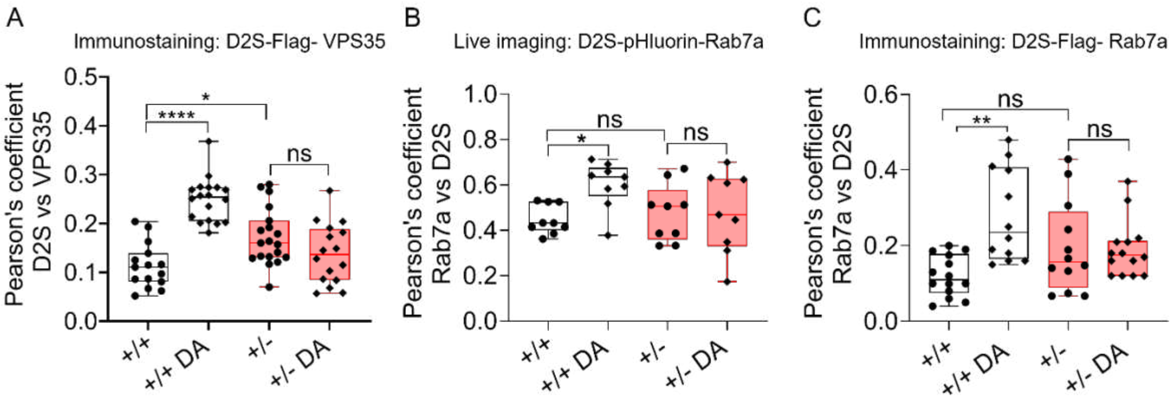

Supplemental Figure 4: Pearson's correlation coefficient colocalization of intracellular D2S with VPS35- and Rab7 positive endosomes. A) Ventral midbrain *Synj1+/+* and *Synj1+/-* neurons expressing FLAG-D2S treated repeatedly with vehicle or 10 μM dopamine, then immunolabeled for anti-VPS35 and anti-FLAG after blocking surface FLAG. Summary of Pearson's correlation coefficient analysis of images from the Figure 7A showing colocalization of iD2S with VPS35. **B-C)** Ventral midbrain *Synj1+/+* and *Synj1+/-* neurons co-expressing pHluorin-D2S and TdTomato-Rab7a **(B)** or co-expressing FLAG-D2S and TdTomato-Rab7a **(C)** were treated repeatedly with vehicle or 10 μM DA, and then either undergo live perfusion of NH_4Cl as in Figure 7D **(B)** or fixed for immunostaining **(C)**. Summary of Pearson's correlation coefficient analysis showing colocalization of iD2S with Rab7a in *Synj1+/+* and *Synj1+/-* neuronal axons. Each data point represents analysis of axons within one field of view. Data from 2 batches of cultures for each condition. *p<0.05, **p<0.01, ****p<0.0001, ns = non-significant, two-way ANOVA with Turkey's multiple comparison.
